# Cilastatin protects against tacrolimus-induced nephrotoxicity via anti-oxidative and anti-apoptotic properties

**DOI:** 10.1186/s12882-019-1399-6

**Published:** 2019-06-14

**Authors:** Kang Luo, Sun Woo Lim, Jian Jin, Long Jin, Hyo Wook Gil, Dai Sig Im, Hyeon Seok Hwang, Chul Woo Yang

**Affiliations:** 1 Convergent Research Consortium for Immunologic Disease, Seoul St. Mary’s Hospital, The College of Medicine, The Catholic University of Korea, Seoul, South Korea; 2 Division of Nephrology, Department of Internal Medicine, Soonchunhyang University Cheonan Hospital, Cheonan, South Korea; 3 Department of Chemistry, College of Natural Sciences, Soonchunhyang University, Asan, South Korea; 4 SH Company, Asan, Chungnam South Korea; 5 Division of Nephrology, Department of Internal Medicine, College of Medicine, Kyung Hee University, Seoul, South Korea; 6 Department of Nephrology, Yanbian University Hospital, Yanbian, China; 7 Division of Nephrology, Department of Internal Medicine, Seoul St. Mary’s Hospital, The Catholic University of Korea, 222 Banpo-daero, Seocho-gu, Seoul, 06591 South Korea

**Keywords:** Cilastatin, Tacrolimus, Nephrotoxicity

## Abstract

**Background:**

Cilastatin (CL) is an inhibitor of dehydropeptidase-I, which is safely used in clinical practice to prevent nephrotoxicity of antibiotics. Tacrolimus (TAC) is the most important immunosuppressant in renal transplantation, but it causes considerable nephrotoxicity. We evaluated the protective effects of CL against chronic TAC-induced nephropathy.

**Methods:**

Chronic nephropathy was induced by administering TAC (1.5 mg/kg/ day, subcutaneous injection) to rats on a low-salt diet for 4 weeks. CL (75 or 150 mg/kg/day, intraperitoneal injection) was concomitantly treated with TAC. Human proximal tubular cells were exposed to TAC (50 μg/mL) with or without CL (250 μg/mL). We investigated the effects of CL on TAC-induced injury in terms of renal function, tubulointerstitial fibrosis, and inflammation. The effects of CL on oxidative stress and apoptosis were evaluated in both in vivo and in vitro models of TAC nephrotoxicity.

**Results:**

CL treatment improved TAC-induced renal dysfunction and decreased renal interstitial fibrosis (reduced expression of e-cadherin and TGFβ-1) and interstitial inflammation (decreased infiltration of ED-1-positive and osteopontin-positive cells). Compared to TAC treatment alone, CL co-treatment reduced oxidative stress (serum 8-OHdG level and immunoreactivity of 8-OHdG and 4-HHE in renal tissue) and increased renal expression of anti-oxidant enzyme, manganese superoxide dismutase. CL treatment decreased apoptotic cell death (decreased TUNEL-positive cells and reduced expression of active caspase-3) in TAC-treated kidney. In vitro CL treatment prevented tubular cell death from TAC treatment and decreased number of annexin V-positive cells were observed in cilastatin-cotreated cells.

**Conclusion:**

CL has protective effects against chronic TAC-induced nephrotoxicity owing to its anti-oxidative and anti-apoptotic properties.

**Electronic supplementary material:**

The online version of this article (10.1186/s12882-019-1399-6) contains supplementary material, which is available to authorized users.

## Background

Tacrolimus (TAC) is a widely used maintenance immunosuppressant, but its long-term use causes considerable nephrotoxicity. TAC causes irreversible kidney injury with arteriolar hyalinization and thickening, vasoconstriction and ischemia, tubulointerstitial fibrosis, apoptosis, and atrophy [1, 2]. The pathogenesis of TAC-induced nephrotoxicity remains unclear, but the direct toxic effects or oxidative stress of TAC on renal cells are suggested as common mechanisms [3, 4].

Cilastatin (CL) is an inhibitor of dehydropeptidase I in the brush border of the renal proximal tubule and is administered in combination with imipenem antibiotics. The role of CL in current clinical practice is to inhibit the formation of renal toxic products by preventing the hydrolysis of imipenem from dehydropeptidase I [5]. Interestingly, several experimental studies have shown that CL reduces the nephrotoxicity of fosfomycin, vancomycin, and cisplatin [6–8]. CL reduces oxidative stress and suppresses apoptotic cell death through modulation of the Fas system [9]. In addition, coadministration of CL does not compromise the efficacy of anti-bacterial and anti-cancer drugs. These findings suggest that CL has its own preventive effects against nephrotoxic drugs and that it is a promising agent for extending the clinical utility of several drugs with nephrotoxicity.

Based on the above findings, we hypothesized that CL may have protective effects against TAC-induced nephrotoxicity. Using an experimental model of chronic TAC-induced nephrotoxicity, we investigated the protective effect of CL against TAC-induced renal toxicity in terms of renal function and histopathology, and also evaluated the possible mechanism of renal protection of CL.

## Methods

### Animals and drugs

The Animal Care and Use Committee of the Catholic University of Korea approved the experimental protocol (CUMC-2016-0167-02), and all procedures performed in this study were in accordance with ethical guidelines for animal studies. Eight-week-old male Sprague Dawley rats (Charles River Technology, Seoul, Korea) that initially weighed 220–230 g were housed in a pathogen-free facility on a 12-h light/dark schedule and with ad libitum access to food and water at the Catholic University of Korea’s animal care facility. Low-salt diet (0.05% sodium, Teklad Premier, Madison, WI, USA) was fed to rats. Tacrolimus (Prograft, Astellas Pharma Inc., Ibaraki, Japan) was dissolved in olive oil (Sigma, St. Louis, MO, USA) at the concentration of 1 mg/mL. CL was obtained from SH company, Asan, Korea.

### Experimental design

After feeding a low-salt diet for 1 week, rats were randomized to six groups containing nine rats. Several numbers are obtained from random number tables. The indicator for randomization is the remainder after the obtained number is divided by six. Rats were treated daily with TAC (1.5 mg/kg, s.c.) or a vehicle (VH, olive oil, 1 mg/ml, s.c.) with or without CL (75 and 150 mg/kg, i.p.) for 4 weeks. The dose and duration of treatment was selected based on previous reports [4, 10, 11].

### Basic protocol

Rats were monitored and treated for 4 weeks. To measure urine volume and water intake over 24 h, metabolic cages (Tecniplast, Gazzada, Italy) were used individually. Animals were anesthetized with Zoletil (30 mg/kg) and Rompun (10 mg/kg) to minimize suffering. When rat movements to pain stimuli were blunted, a 3 ml syringe was inserted into the internal jugular vein to take blood samples. Animals were euthanized via CO_2_ narcosis after sample collection. Kidney cortex tissue was homogenized in lysis buffer. Homogenates were centrifuged at 3000 rpm for 15 min, and Bradford method (Bio-RAD, Hercules, California, United States) was used to determine protein concentration. Kidney tissues were also fixed in a periodate-lysine-paraformaldehyde solution and embedded in wax. After dewaxing, 4-μm sections were processed for further analysis. Urinary excretion of albumin was examined at Samkwang Medical Laboratories (Seoul, Korea) using enzymatic colorimetric methods (Modular DPP system, Roche, Hamburg, Germany). All histologic and immunoblot analysis were examined in blind condition to each group information. The quantitative enzyme colorimetric method (Stanbio Laboratory, Boerne, TX, USA) was used to measure serum creatinine (Scr) and blood urea nitrogen (BUN). Creatinine clearance (CrCl) was calculated using 24 h urine collection and serum creatinine. The TAC concentration in whole blood and kidney was measured using liquid chromatography-tandem mass spectrometry and a General Tacrolimus ELISA Kit (E1207Ge; EIAab Science, Wuhan, China), respectively.

### Measurement of interstitial fibrosis in kidney

Histological assessment was defined as the development of tubule interstitial fibrosis in trichrome-stained tissue sections, as described previously [12]. Matrix-richexpansion of the interstitium with tubular dilatation, atrophy, and cast formation was primarily defined as tubulointerstitial fibrosis. Epithelial cell sloughing or basement membrane thickening in tubular cells were also considered as tubulointerstitial fibrosis. The extent of fibrosis was estimated by counting the percentage of injured areas per field using a polygon program with a color image analyzer (TDI Scope Eye Version 3.0 for Windows; Seoul, Korea).

### Immunohistochemistry

Immunohistochemistry (IHC) was performed as described previously [13, 14]. Immunoreactive cells were detected in 4 μm tissue sections. They were incubated with specific antibodies against ED-1 (AbD Serotec, Oxford, UK), osteopontin (OPN, obtained from the Developmental Studies hybridoma bank, University of Iowa, Iowa City, IA, USA), 8-hydroxy-2′-deoxyguanosine (8-OHdG, JaICA, Shizuoka, Japan), 4-hydroxy-2-hexenal (4-HHE, JaICA), and active caspase-3 (Millipore, St. Charles, MO, USA) for 12 h at 4 °C. Each section were examined in 20 fields at 200× magnification using the color image analyzer (TDI Scope Eye).

### Immunoblot analysis

From tissue lysates from the renal cortex, e-cadherin (BD Biosciences, San Jose, CA, USA), TGFβ-1 (R&D Systems, Minneapolis, MN, USA), MnSOD (Abcam, Cambridge, UK), and β-actin (Sigma) were detected by incubating for 12 h with specific antibodies [15]. Image analyzer (Quantity One version 4.4.0; Bio-Rad, Hercules, CA, USA) were used to analyze the immunoblot.

### In situ terminal deoxynucleotidyl transferase-mediated dUTP–biotin nick end labeling (TUNEL) assay

To identify apoptosis in tissue sections, the ApopTag In Situ Apoptosis Detection Kit (Millipore) was used. The number of TUNEL-positive cells was counted in 20 fields at 200× magnification.

### Measurement of serum and urine 8-OHdG level

To detect the DNA adduct 8-OHdG as marker of oxidative DNA damage, competitive enzyme-linked immunosorbent assay (Cell Biolabs, San Diego, CA, USA) was used in the serum and 24 h urine.

### Measurement of cell viability

Human kidney-2 (HK-2) cells was purchased from the American Type Cell Collection and were seeded into 96-well plates at a density of 2.5 × 10^4^ and were pre-incubated for 24 h in an incubator at 37 °C. After 12 h, the culture media was replaced to serum-free media with TAC (50 μg/mL) and CL (250 μg/mL). Cell viability was determined using a Cell Counting Kit-8 assay kit (Dojin Laboratories, Kumamoto, Japan).

### Flow cytometry

Flow cytometry was performed to assess fluorescein isothiocyanate (FITC)-conjugated annexin V (BD Biosciences) production. HK-2 cells were seeded into six-well plates at a density of 2.5 × 10^5^ cells/well and pre-incubated for 12 h at 37 °C in an incubator. Trypsinized cells were treated with 5 μL of FITC- conjugated annexin V (BD Biosciences) in 1× binding buffer (BD Biosciences) for 15 min at room temperature according to the manufacturer’s protocol. Values are expressed as the percentage of fluorescent cells relative to the total cell count.

### Statistical analysis

The data are expressed as means ± SEM of at least three independent experiments. We examined the normality test in all variables using Shapiro-Wilk test (Additional file 1: Table S1–3). Multiple comparisons between groups were performed by one-way ANOVA with the Bonferroni post hoc test, if variables showed normal distrubution (SPSS software version 19.0; IBM, Armonk, NY, USA). Kruskal–Wallis test or Mann-Whitney*U* test was used in case of variables without normal distribution. Statistical significance was assumed as *P* < 0.05.

We performed power calculations to estimate the sample size using standard formulas (*α* error = 0.05; *β* error = 0.2). The sample size required to show 6% difference in tubulointerstitial fibrosis between TAC and TAC + CL75 was calculated. The minimal number of rats was nine, if we considered 20% of drop rate.

## Results

### Effect of CL on TAC-induced renal dysfunction

All animals were analyzed to investigate the effect of cilastatin on TAC-induced nephrotoxicity. Table 1 lists the changes in functional basic parameters in the control and experimental groups. The TAC, TAC + CL75, and TAC + CL150 groups showed lower body weight gain than that by the VH, VH + CL75, and VH + CL150 groups. The TAC-treated group with or without CL had a greater urine volume and larger water intake than the control group. The levels of Scr and BUN and the amount of microalbuminuria were significantly higher in the TAC group than in the control group. Cotreatment with CL reverted these changes. CL treatment improved the rate of CrCl, which was decreased in the TAC group. CL did not affect the trough level of TAC in the whole blood and kidney tissues.

**Table 1:** Effect of CL on basic parameters and TAC concentration

	VH	VH + CL75	VH + CL150	TAC	TAC + CL75	TAC + CL150
△BW (g)	79 ± 5	77 ± 9	83 ± 5	59 ± 6^#^	48 ± 6^#^	48 ± 4^#^
UV (mL/day)	9 ± 2	13 ± 1	25 ± 1^#^	26 ± 3^#^	31 ± 7^#^	22 ± 3^#^
WI (mL/day)	15 ± 3	18 ± 3	30 ± 3^#^	30 ± 3^#^	34 ± 7^#^	26 ± 3^#^
Scr (mg/dL)	0.35 ± 0.07	0.32 ± 0.02	0.32 ± 0.03	0.64 ± 0.05^#^	0.49 ± 0.02^@^	0.38 ± 0.05^@^
BUN (mg/dL)	14.1 ± 3.1	14 ± 1.4	14.2 ± 0.7	56.2 ± 6.9^#^	45.5 ± 1.4^@^	42.9 ± 4.5^@^
UMA (μg/mg)	1.6 ± 0.2	2.3 ± 0.2	2.4 ± 0.2	9.2 ± 1.2^#^	7.7 ± 1.3	4.8 ± 1.0^@^
CrCl (mL/min/100 g)	0.77 ± 0.13	0.76 ± 0.06	0.81 ± 0.11	0.35 ± 0.02^#^	0.50 ± 0.03^@^	0.62 ± 0.08^@^
TAC con.in blood (ng/mL)	_	_	_	14.1 ± 2.4	13.5 ± 2.1	13.9 ± 1.9
TAC con.in kidney (pg/mg)	_	_	_	7.4 ± 1.3	6.3 ± 1.1	5.1 ± 1.6

^#^*P* < 0.05 vs. VH

^@^*P* < 0.05 vs. TAC

*BW* body weight, *UV* urine volume, *WI* Water intake, *Scr* serum creatinine, *BUN* blood urea nitrogen, *UMA* urine microalbumin, *CrCl* creatinine clearance, *TAC con.* tacrolimus concentration, *VH* vehicle, *TAC* tacrolimus; CL75 and CL150, 75 and 150 mg/kg of CL values are means ± standard error

### Effect of CL on fibrosis in TAC-treated kidney

**Fig. 1:**
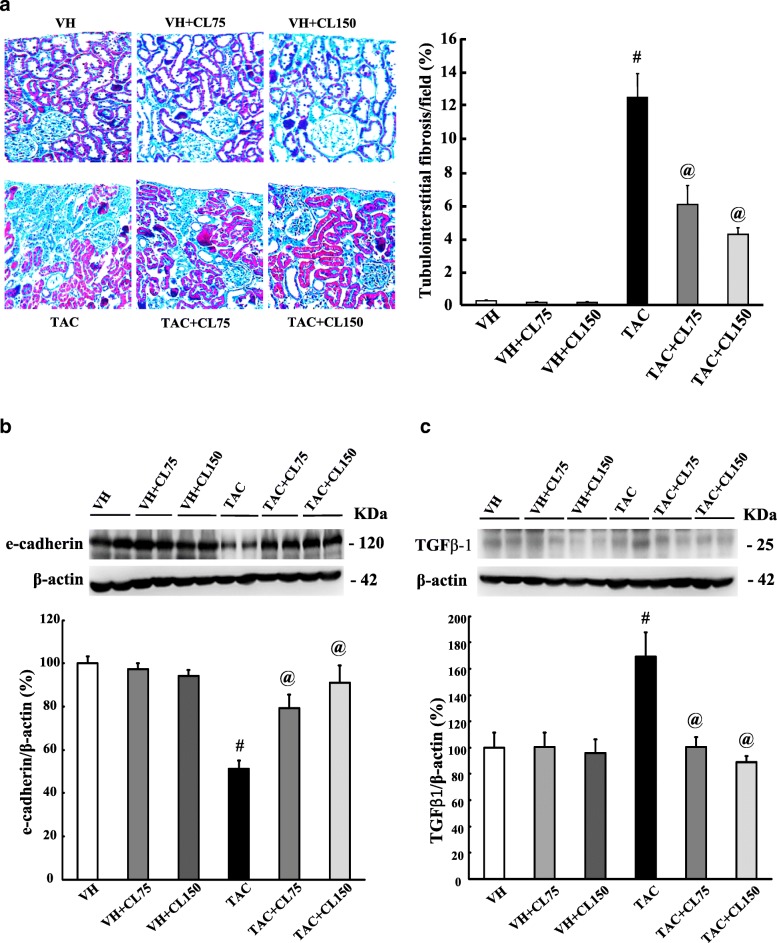
Effect of CL on fibrosis during TAC-induced renal injury. **a** Histological analysis of renal cortex treated with TAC shows striped tubulointerstitial fibrosis, mononuclear cell infiltration, and tubular atrophy. CL treatment significantly reduced these damages as compared with TAC treatment. (**b** and **c**) Immunoblot analysis of e-cadherin and transforming growth factor β1 (TGFβ-1) in the renal cortex. Note that combined treatment with CL reversed changes of e-cadherin and TGFβ-1 in response to TAC treatment. Magnification, 200×. *N* = 9 per group. ^#^*P* < 0.05 vs. VH; ^@^*P* < 0.05 vs. TAC

TAC treatment induced extensive interstitial fibrosis, and CL treatment significantly reduced interstitial fibrosis (Fig. 1a). TAC treatment decreased the e-cadherin expression and increased those of TGFβ-1; CL treatment offset these changes of expression levels in TAC-treated kidney (Fig. 1b, c and Additional file 2: Figure S1 and 2).

### Effect of CL on inflammation in TAC-treated kidney

**Fig. 2:**
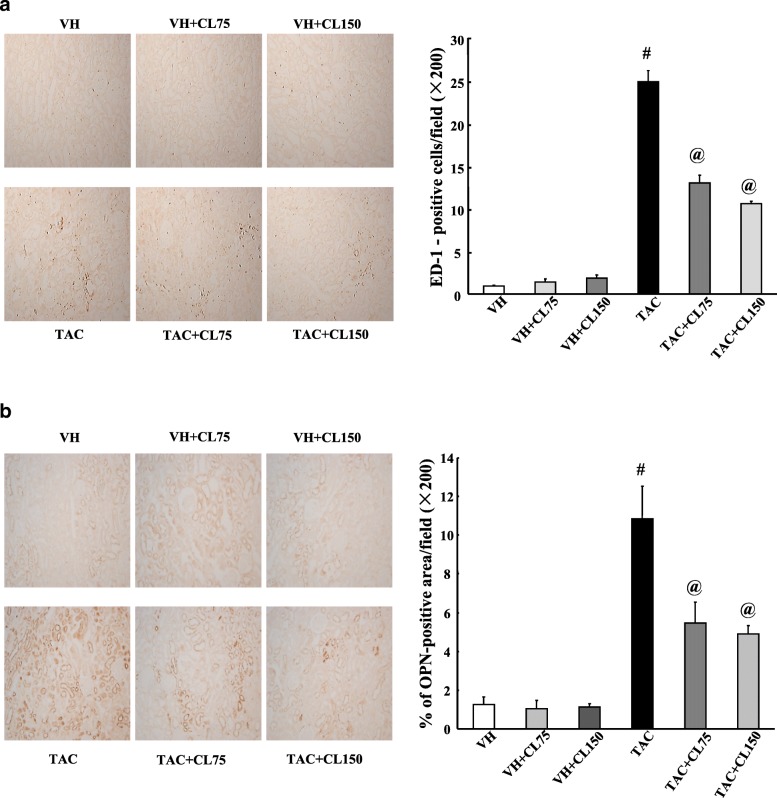
Effect of CL on inflammation during TAC-induced renal injury. **a** and **b** Immunohistochemistry and quantitative analysis for ED-1 and osteopontin (OPN) in the renal cortex. Note that the number of ED-1-positive cells and the intensity of OPN staining are increased in the TAC group and that this increase is inhibited in the TAC + CL groups. Magnification, 200×. N = 9 per group. ^#^*P* < 0.05 vs. VH; ^@^*P* < 0.05 vs. TAC

We investigated the infiltration of ED-1-positive cells and expression of OPN in kidney tissues to find out the effect of CL on inflammatory process. There were rare ED-1-positive cells in the VH and VH + CL groups (Fig. 2a). However, these numbers were significantly increased after TAC treatment, and cotreatment with CL markedly attenuated the infiltration of ED-1 positive cells. Consistently, OPN expression was enhanced in the TAC group, and it was decreased after CL treatment (Fig. 2b).

### Effect of CL on oxidative stress in TAC-treated kidney

**Fig. 3:**
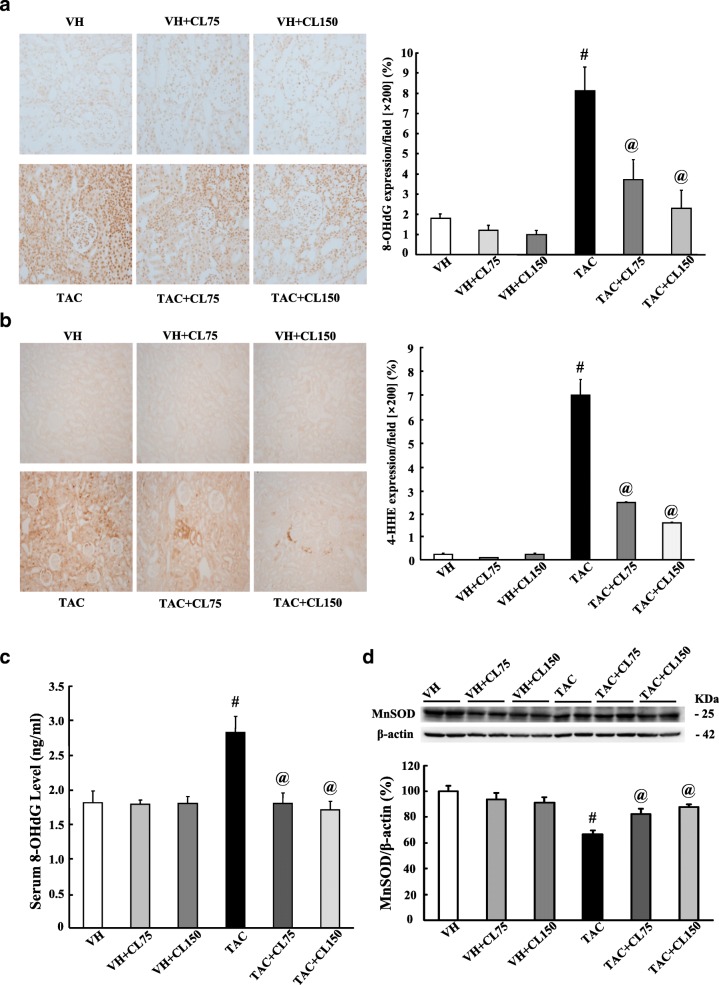
Effect of CL on oxidative stress during TAC-induced renal injury. **a** and **b** Immunohistochemistry for 8-hydroxy-2′-deoxyguanosine (8-OHdG) and 4-hydroxy-2-hexenal (4-HHE) in tissue sections. Intense nuclear expression of 8-OHdG and 4-HHE is shown in the TAC group, and administration of CL reduced their expression levels. **c** Enzyme-linked immunosorbent assay of serum 8-OHdG levels. Serum 8-OHdG was decreased in the CL-cotreated groups as compared to those in the TAC group. **d** Immunoblot analysis of manganese superoxide dismutase (MnSOD) in the renal cortex. Note that combined treatment with CL restored their expression as compared with TAC treatment alone. Magnification, 200×. *N* = 9 per group. ^#^*P* < 0.05 vs. VH; ^@^*P* < 0.05 vs. TAC

Figure 3a and b demonstrated the results of immunohistochemistry of 8-OHdG and 4-HHE, which is a marker of oxidative DNA damage. The strong nuclear expression of 8-OHdG and 4-HHE was observed in the TAC group, and these adverse effects were reduced after CL treatment. The serum 8-OHdG level was much higher in the TAC group than in the VH group, and CL administration considerably decreased serum 8-OHdG levels (Fig. 3c). We also evaluated the expression of the antioxidative molecule MnSOD. The expression of MnSOD was decreased in the TAC group as compared with the control groups, and CL treatment recovered its expression (Fig. 3d and Additional file 2: Figure S3).

### Effect of CL on apoptosis in TAC-treated kidney

**Fig. 4:**
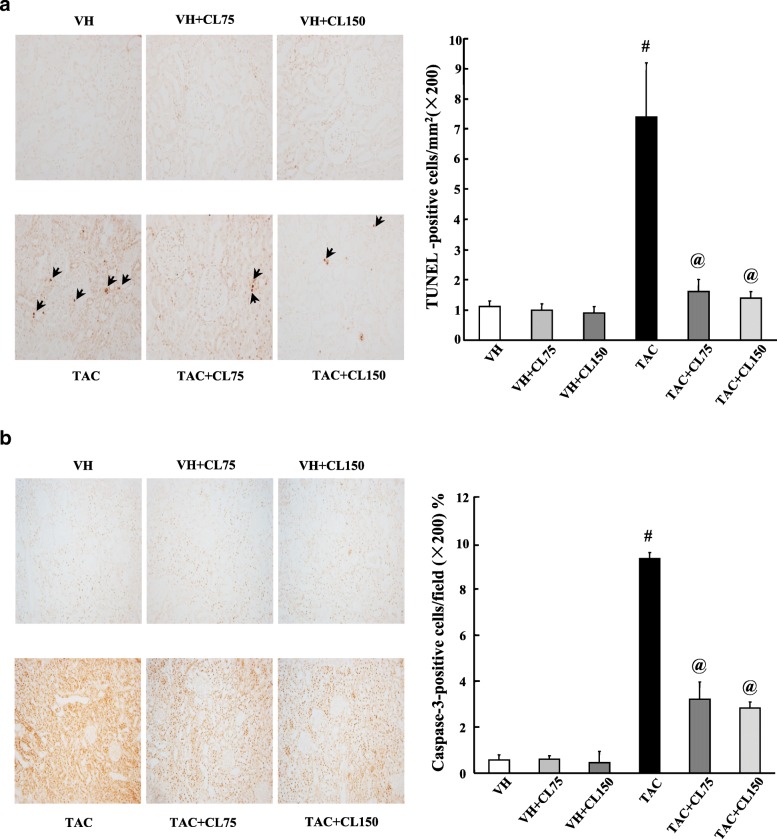
Effect of CL on apoptosis during TAC-induced renal injury. **a** In situ TdT-mediated dUTP-biotin nick end labeling (TUNEL) assay and its analysis in the renal tissue sections. CL cotreatment significantly reduced TUNEL-positive cells as compared to TAC treatment. **b** Representative images of immunohistochemical staining for the active form of caspase-3 (caspase-3) in kidney tissues. Quantitative analysis of caspase-3 in kidney tissues showed that CL cotreatment significantly reduced active caspase-3 staining as compared to TAC treatment alone. The arrows indicate TUNEL-positive apoptotic bodies. Magnification, 200×. *N* = 9 per group. ^#^*P* < 0.05 vs. VH; ^@^*P* < 0.05 vs. TAC

We evaluated the effects of CL treatment on apoptosis. Greater number of TUNEL-positive cells was observed in the TAC group as compared to those of VH group, and the addition of CL reduced these changes (Fig. 4a). CL cotreatment also exhibited decrements in the active form of caspase-3 in kidney tissues as compared with TAC treatment alone (Fig. 4b).

### Effects of CL on proximal tubular cells with TAC treatment

**Fig. 5:**
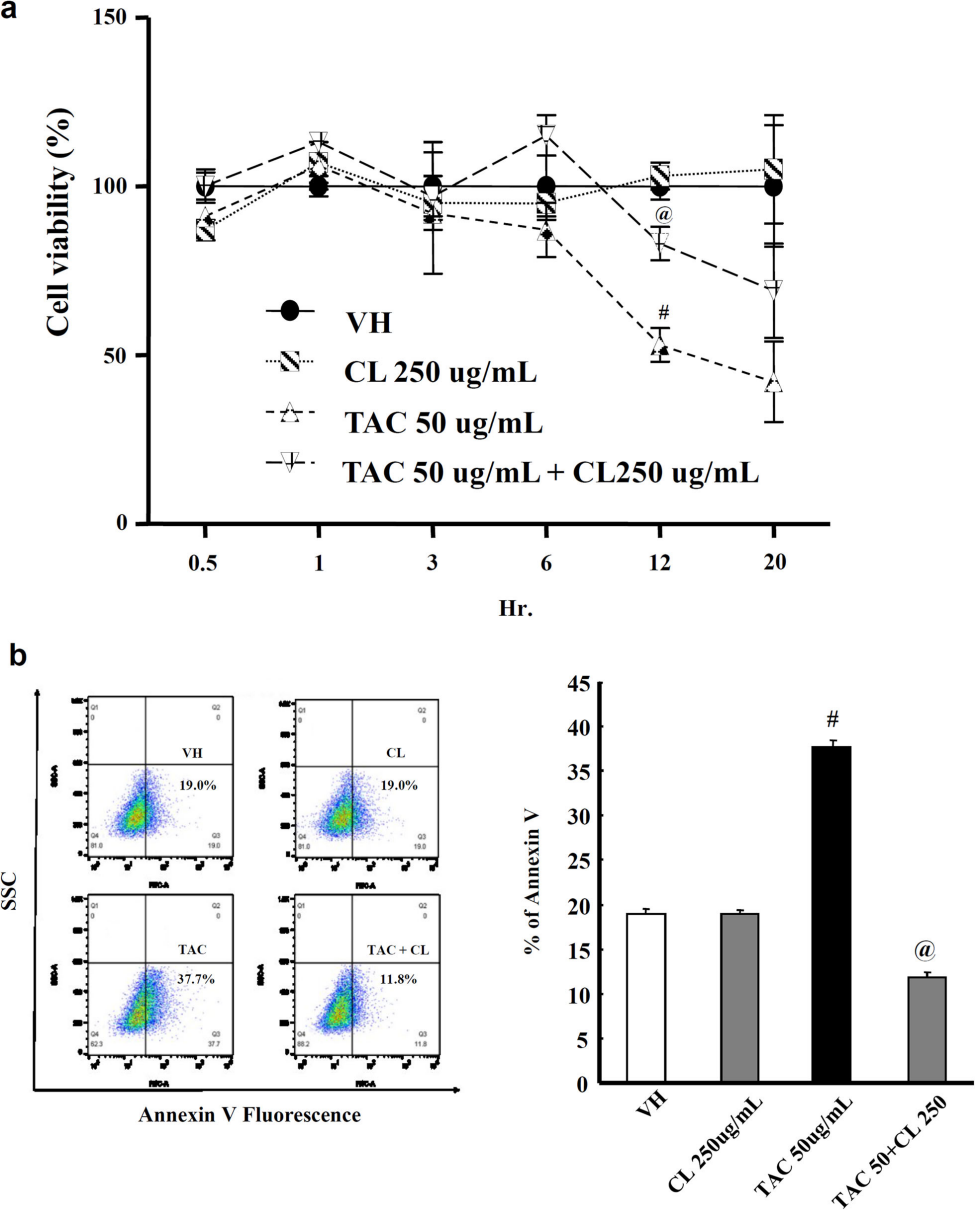
Effect of CL on proximal tubular cells with TAC treatment. **a** Viability of HK-2 cells in TAC treatment with or without CL cotreatment. Note that CL cotreatment improved cell viability in TAC-treated cells. **b** Evaluation of apoptosis using FITC–annexin V staining followed by flow cytometry and graphing showed that the mean percentage of annexin V-positive cells was decreased in the TAC + CL group as compared to the TAC group. ^#^*P* < 0.05 vs. VH; ^@^*P* < 0.05 vs. TAC

The viability of HK-2 cells was significantly decreased in the TAC group as compared to the VH group, and CL increased the viability of TAC-treated cells (Fig. 5a). To evaluate the protective effect of CL on apoptosis, we measured the binding activity of FITC-annexin V. TAC treatment increased the number of FITC-annexin V binding cells as compared to the VH group, and CL treatment significantly decreased annexin V positive cells (Fig. 5b).

## Discussion

The present study was undertaken to investigate whether CL has a protective effect against TAC-induced nephropathy. The results clearly demonstrate that CL treatment improved not only TAC-induced renal dysfunction and proteinuria but also histopathology (decreased interstitial fibrosis and inflammation). In addition, we found that CL treatment effectively suppressed the main pathogenetic mechanism of TAC renal injury (oxidative stress and apoptotic cell death). These findings suggest that addition of CL is useful to reduce TAC-induced renal injury in clinical practice.

Renal interstitial fibrosis is a typical histological feature of TAC-induced nephrotoxicity. While previous studies have shown the protective effects of CL against drug-induced inflammation or apoptosis [9, 16], to our knowledge, there are no reports investigating the effect of CL on renal fibrosis. Using an experimental model of chronic TAC-induced nephrotoxicity, we demonstrated that CL treatment decreased the fibrotic area within the renal interstitium. At the molecular level, the expression of profibrotic cytokine TGFβ-1 was decreased and expression of e-cadherin was recovered in CL-treated rats in a dose-dependent manner. These findings suggest that CL effectively suppresses the progression of fibrosis from TAC-induced renal injury.

Inflammation is reported to be involved in the development and amplification of calcineurin inhibitor (CNI)-induced fibrosis [17]. In previous studies, we demonstrated that TAC treatment induces the infiltration of inflammatory cells and the production of proinflammatory cytokines [18, 19]. Therefore, we investigated whether CL affects TAC-induced macrophage infiltration and inflammatory factors. The administration of CL reduced the amount and immunoreactivity of ED-1, as well as levels of OPN expression. These findings suggest that CL inhibits TAC-induced inflammation by inhibiting macrophage infiltration and by suppression of inflammatory mediators.

Oxidative stress and production of ROS is a common pathway in TAC-induced renal injury [20, 21], and inflammation and oxidative stress are central mechanisms leading to apoptotic cell death [22–24]. We previously reported that TAC-induced oxidative stress induces apoptotic and autophagic cell death, and this is closely associated with structural and functional kidney injury [13, 25, 26]. In the current study, CL treatment recovered the antioxidant (MnSOD) and reduced the expression of 8-OHdG and 4-HHE in TAC-induced renal injury. These findings confirm that CL decreases TAC-induced oxidative stress. We further evaluated the effect of CL treatment on apoptosis in TAC-injured kidneys, and found that that CL significantly reduced the number of TUNEL-positive cells and caspase-3 expression. This result was consistent with our previous report that CL has anti-apoptotic effects in vancomycin-induced nephrotoxicity [11].

While we demonstrated the renoprotective effects of CL in vivo, it is still undetermined whether CL has direct effects on renal tubular cells. Therefore, we evaluated the effect of CL on HK-2 cells, a main target site for TAC injury [27], and found that CL decreased TAC-induced cell death and the number of FITC-conjugated Annexin V-positive cells as compared with TAC alone. These findings suggest that CL has a direct effect of protecting proximal tubular cells in the setting of TAC-induced nephrotoxicity.

The results of this study clearly demonstrate that CL has a protective effect against TAC-induced renal injury, but our study had some limitations. First, our experimental model of chronic TAC nephropathy was induced in rat. There might be species specificity. Second, we did not measure TAC levels in renal tubular cells. Therefore, we could not evaluate the effect of CL on TAC levels in renal tubular cells. Third, we did not include drug interactions between CL and TAC. Pharmacokinetic studies might be needed to determine whether CL affects blood TAC levels. Finally, we found a greater urine volume and larger water intake in VH + CL150 than in VH group. We could not know the exact reason in this study, but presume that CL also affects the water balance in kidney or intestine, which has active site of CL. Further studies are needed to explore the effect of CL on water reabsorption.

## Conclusions

CL has protective effects on TAC-induced nephrotoxicity. Anti-oxidative and anti-apoptotic properties are associated with protective effects of CL in TAC-induced nephrotoxicity. Our study provides the rationale for the clinical usefulness of CL to prevent TAC-induced nephrotoxicity, and randomized clinical trials are needed to evaluate the protective effect of CL in TAC-based immunosuppression.

## Additional files

Additional file 1: **Table S1-S3.***P* value of normality test in experimental data. *P* value of normality test in basic parameters, TAC concentration, animal and cell experimental variables. (XLSX 22 kb)

Additional file 2: **Figure S1-S3.**Full images of western blot. Immunoblot images including molecular size markers of Fig. 1b, c and 3d). (PPTX 18383 kb)

## Data Availability

The datasets used and/or analysed during the current study are available from the corresponding author on reasonable request.

## Abbreviations

4-HHE: 4-hydroxy-2-hexenal
8-OHdG: 8-hydroxy-2`-deoxyguanosine
BUN: Blood urea nitrogen
CL: Cilastatin
Clcr: Creatinine clearance
i.p.: Intraperitoneal injection
IHC: Immunohistochemistry
MnSOD: Manganese superoxide dismutase
s.c.: Subcutaneously
Scr: Serum creatinine
TAC: Tacrolimus
TGFβ-1: Transforming growth factor beta-1
TUNEL: Terminal deoxynucleotidyl transferase-mediated dUTP nick-end labeling
VH: Vehicle
