# Supplementary material for: Cilastatin protects against tacrolimus-induced nephrotoxicity via anti-oxidative and anti-apoptotic properties
Source: BMC Nephrol. 2019 Jun 14;20:221. doi: 10.1186/s12882-019-1399-6 (PMC6570925; doi:10.1186/s12882-019-1399-6)
Supplement: Supplementary file 2 — Figure S1-S3.Full images of western blot. Immunoblot images including molecular size markers of Fig. 1b, c and 3d). (PPTX 18383 kb) [file 12882_2019_1399_MOESM2_ESM.pptx]

## Slide 1
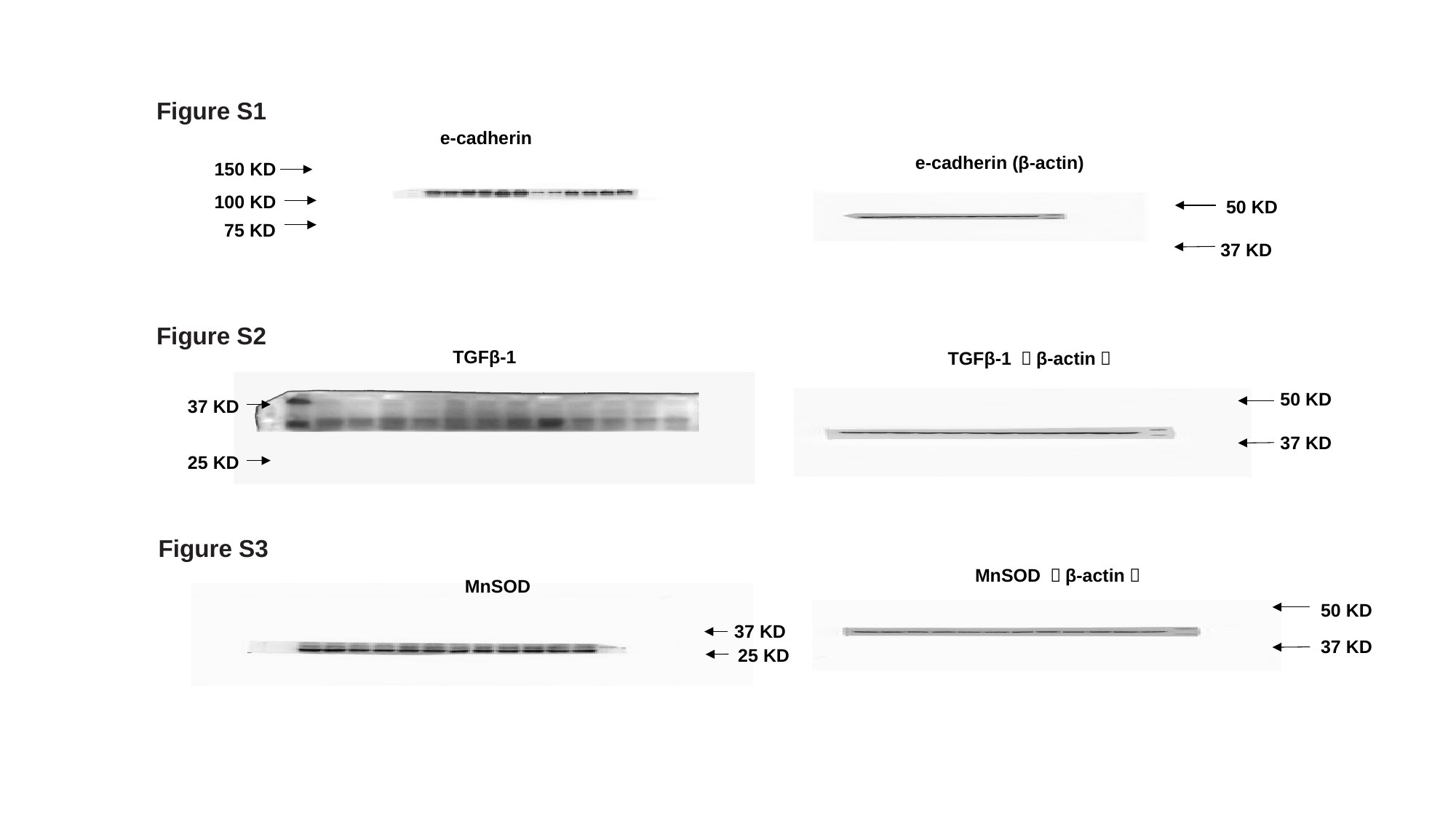

Figure S1
e-cadherin
150 KD
100 KD
75 KD
e-cadherin (β-actin)
50 KD
37 KD
Figure S2
TGFβ-1
37 KD
25 KD
TGFβ-1 （β-actin）
50 KD
37 KD
Figure S3
MnSOD （β-actin）
50 KD
37 KD
MnSOD
37 KD
25 KD
